# Supplementary material for: Disruption of auto-inhibition underlies conformational signaling of ASIC1a to induce neuronal necroptosis
Source: Nat Commun. 2020 Jan 24;11:475. doi: 10.1038/s41467-019-13873-0 (PMC6981194; doi:10.1038/s41467-019-13873-0)
Supplement: Supplementary file 1 — Supplementary Information [file 41467_2019_13873_MOESM1_ESM.pdf]

Supplementary Figure 1 by Wang *et al.*

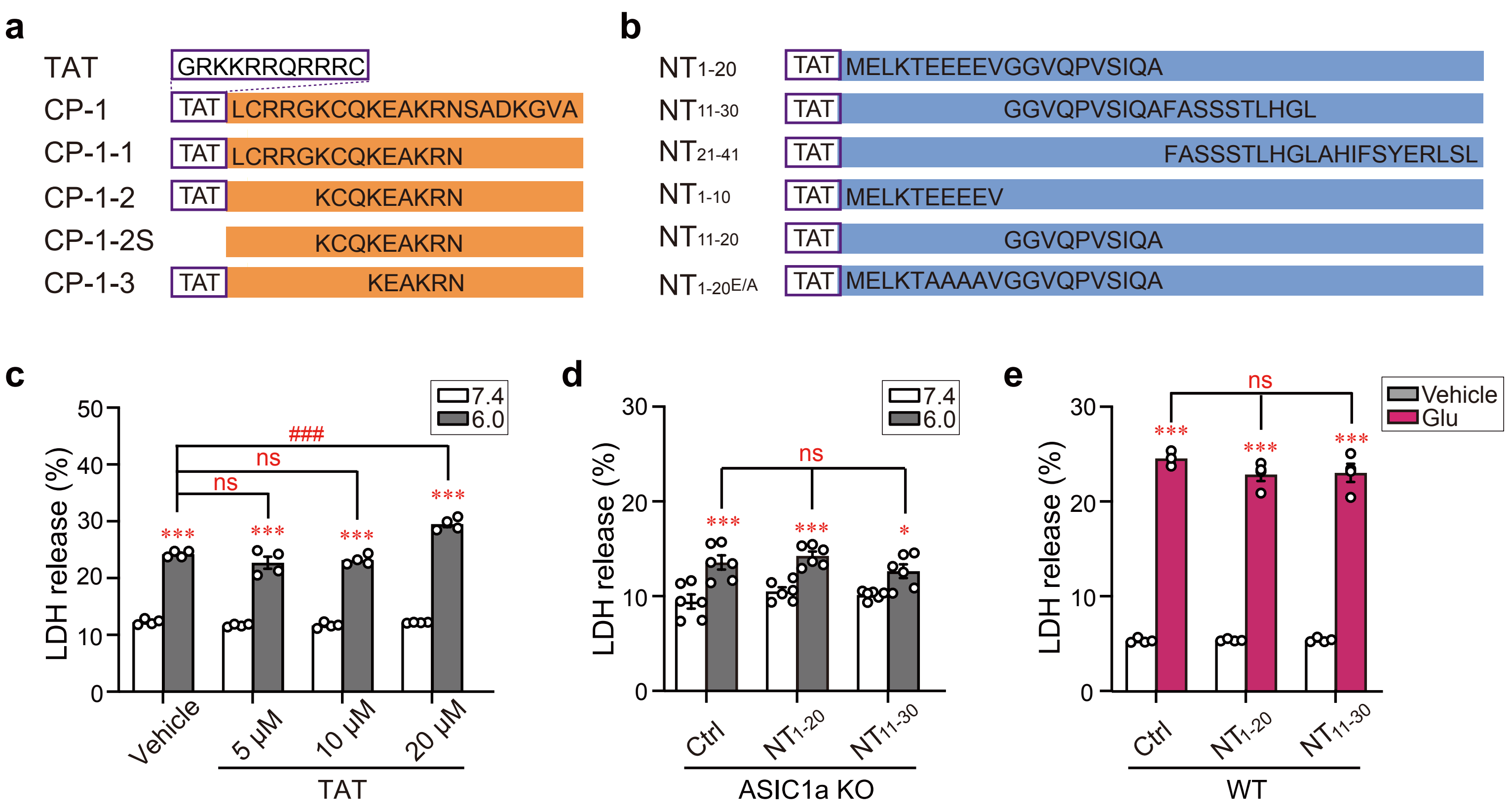

Supplementary Figure 1. Design and characterization of ASIC1a-derived peptides. (**a**) Sequences of membrane-penetrating TAT peptide and ASIC1a C-terminal derived peptides. (**b**) Sequences of ASIC1a N-terminal derived peptides. (**c**) High concentration (20 μM) of TAT (Ctrl) peptide increased acid-induced LDH release in cultured cortical neurons. n = 4, *** p <0.001 vs. corresponding pH 7.4, ns, no statistical significance, ### p <0.001 vs. vehicle (Veh) in pH 6.0, by ANOVA. (**d**) In contrast to wild type neurons, NT peptides had no effect on acid (pH 6.0)-induced death of cortical neurons prepared from ASIC1a knockout (KO) mice. LDH assay, n = 6, *p <0.05, ***p <0.001 vs. corresponding pH 7.4 treatment, ns, no statistical significance vs. Ctrl in pH 6.0, by ANOVA. (**e**) Peptides NT_{1-20} and NT_{11-30} did not protect neurons against excitatoxicity from glutamate (Glu, 10 μM, 0.5 hr) in cultured neurons. LDH assay, n = 3–4, ***p <0.001 vs. the same condition with Vehicle, ns, no statistical significance vs. Ctrl with glutamate treatment, by ANOVA.

Supplementary Figure 2 by Wang *et al.*

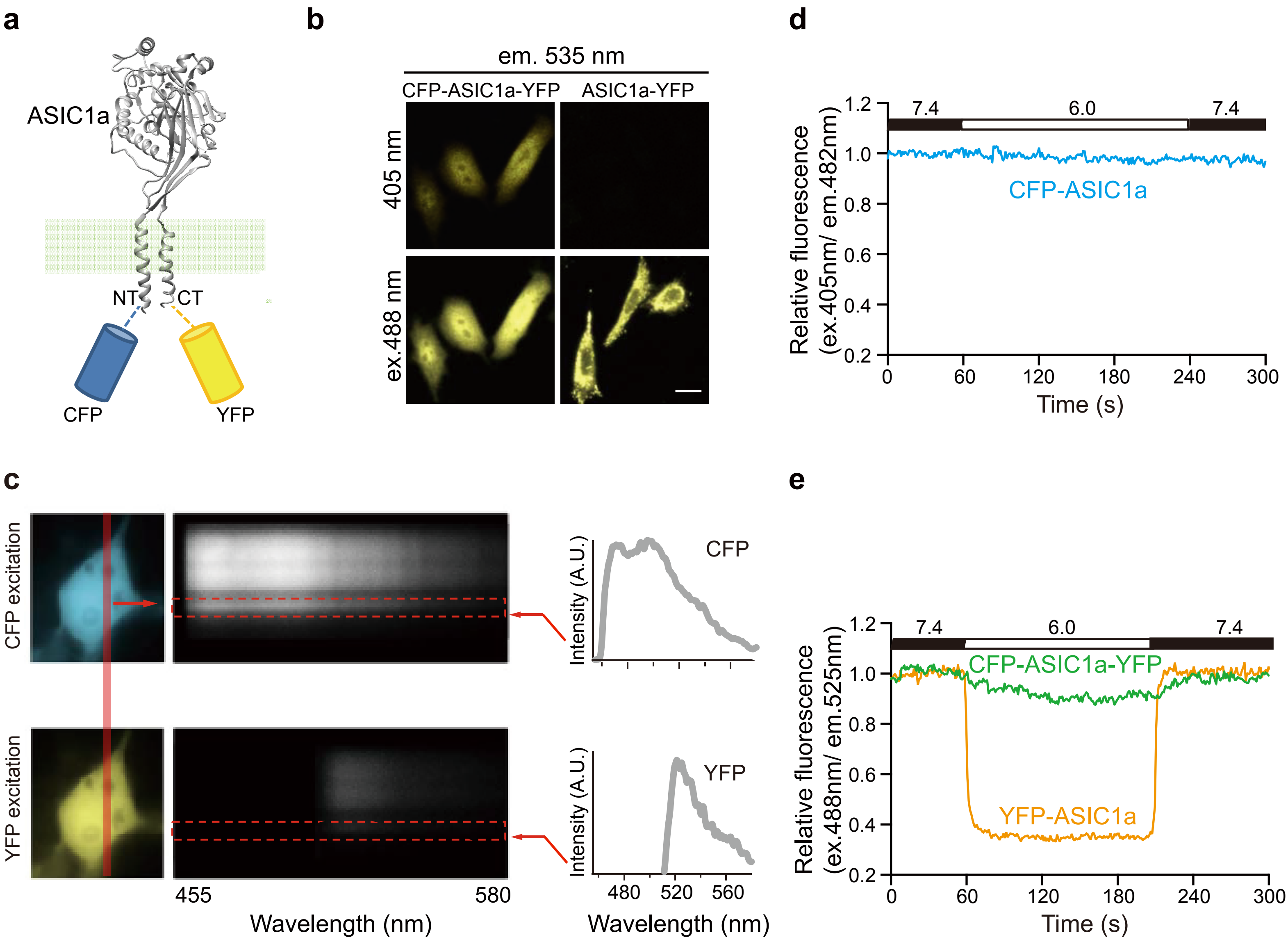

Supplementary Figure 2. FRET protocols. (**a**) The model of ASIC1a tagged at its C- and N- termini with YFP and CFP, respectively. (**b**) YFP emission images in response to different excitation wavelengths in transfected CHO cells that expressed CFP-ASIC1a-YFP or ASIC1a-YFP. (**c**) The protocol to measure the FRET efficiency with spectra FRET. A single cell transfected with CFP–ASIC1a-YFP was shown with CFP or YFP excitation light (left). Spectroscopic images from the cellular region under the slit (indicated by rectangles) are shown, in which the y axis represents the position of the cell, the x axis represents the wavelength (middle). Using line-scanning measurement of the fluorescence intensity values along the upper membrane region (red arrows) of the spectroscopic images, the emission spectra of CFP and YFP are constructed (right). (**d**) The fluorescence intensity of CFP-ASIC1a (405 nm excitation, 482 nm emission) did not change much in response to pH 6.0 treatment. (**e**) pH 6.0 treatment drastically reduced the fluorescence intensity of ASIC1a-YFP, but caused only moderate fluorescence intensity decrease of YFP in CFP-ASIC1a-YFP (488 nm excitation and 525 nm emission).

Supplementary Figure 3 by Wang *et al.*

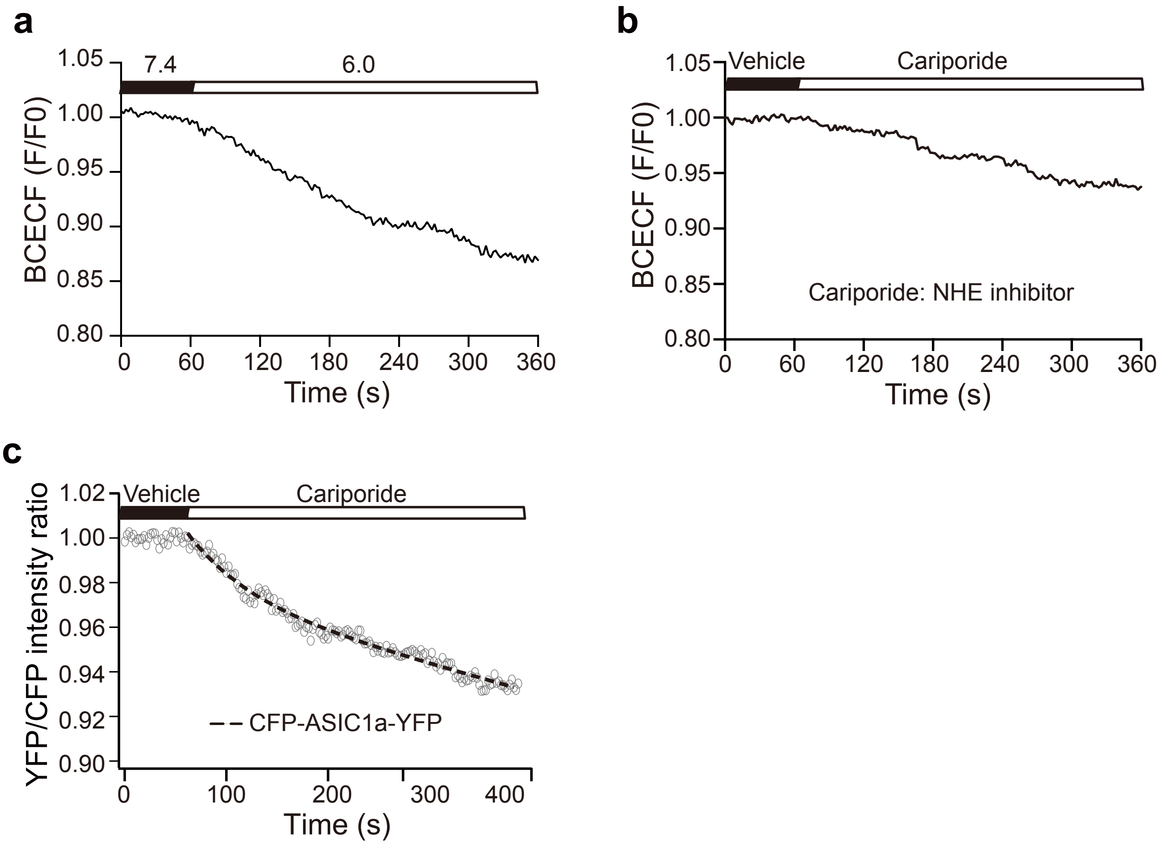

Supplementary Figure 3. Intracellular acidification measured by BCECF. (**a, b**) CHO cells were loaded with BCECF and intracellular pH changes were monitored in response to switching the extra-cellular pH from 7.4 to 6.0 (**a**) or treatment with the Na+/H+ exchanger (NHE) blocker, cariporide (10 μM, **b**), as indicated. Both treatments caused slow decreases in intracellular pH. Note, the global intracellular pH reduction caused by extracellular acidification, as indicated by BCECF, was much slower than the near membrane cytoplasmic pH drop demonstrated by ASIC1a-YFP (Supplementary Figure 2e). (**c**) Intracellular acidification induced by inhibiting NHE caused dissociation of ASIC1a NT from CT. Cells expressing CFP-ASIC1a-YFP were treated with cariporide (10 μM) while changes in YFP/CFP ratio were monitored as in Fig. 2b (F525/F482 nm emission with 405 nm excitation). The FRET decrease paralleled the decrease in intracellular pH induced by cariporide (**b**). This could suggest that intracellular acidification alone induced the dissociation of ASIC1a-NT from its CT. However, it cannot be ruled out if under these experimental conditions, the cariporide treatment might have also affected the local pH near the extracellular domains of ASIC1a.

Supplementary Figure 4 by Wang *et al.*

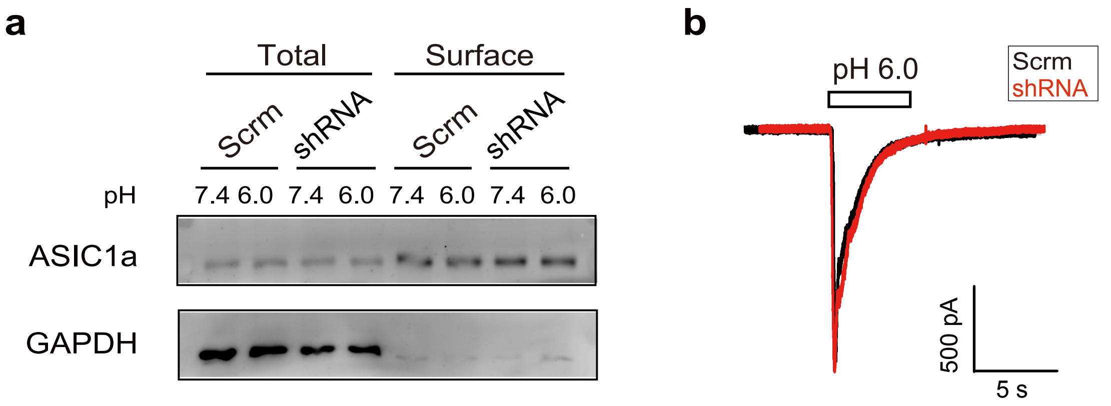

Supplementary Figure 4. NSF knockdown by shRNA has no effect on the surface expression of ASIC1a or ASIC1a-mediated currents. (**a**) Representative western blots for surface and total expression of ASIC1a in neurons transfected with scrambled (Scrm) and NSF shRNA (shRNA). (**b**) Representative pH 6.0-induced current traces at -60 mV of cultured neurons transfected with Scrm and NSF shRNA.

Supplementary Figure 5 by Wang *et al.*

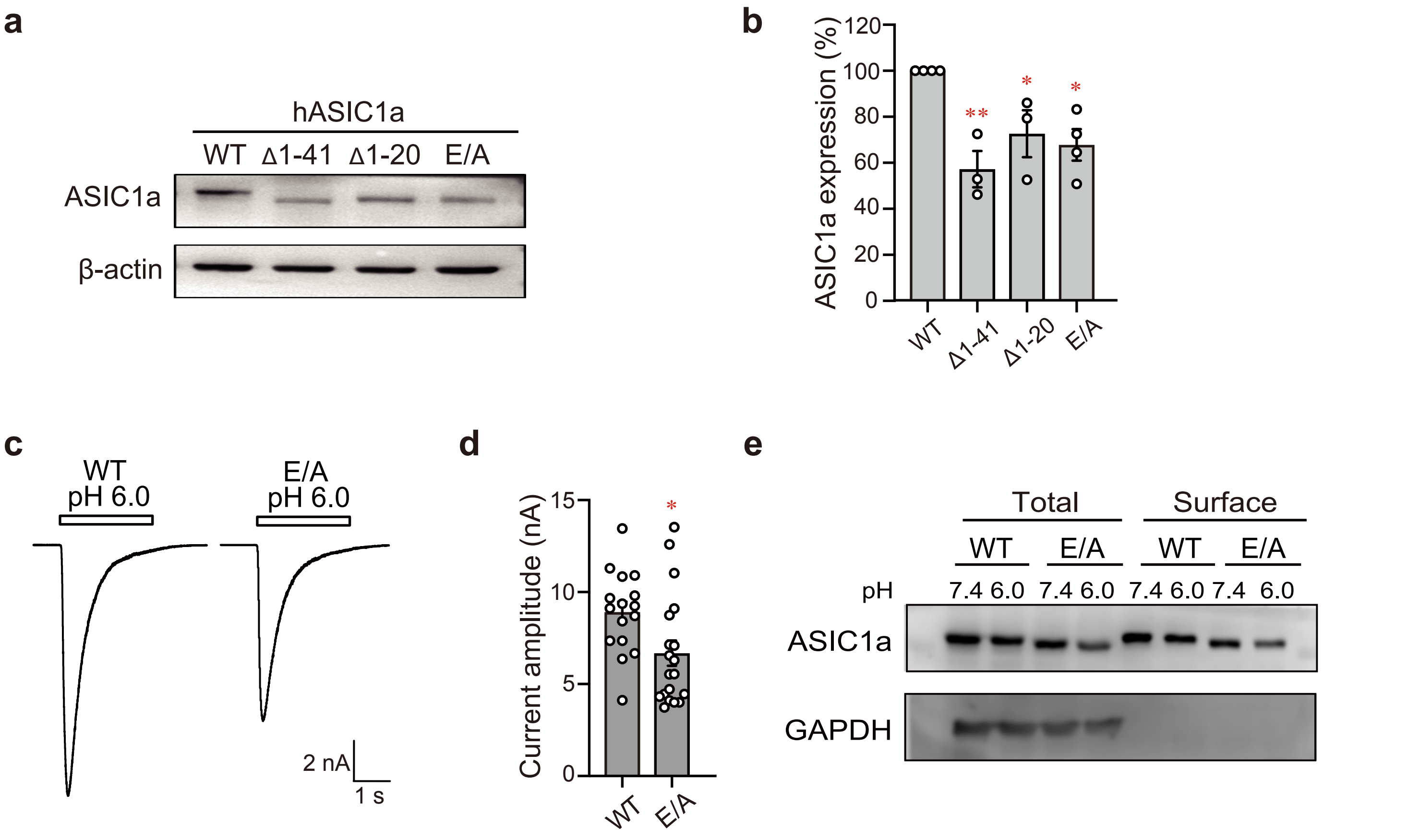

Supplementary Figure 5. NT deletions and E^6EEE^9 to AAAA mutation result in reduced expression of ASIC1a. ASIC1a NT deletion and E/A mutants were transfected into CHO cells and cultured in normal medium at pH 7.4. Cell lysates were subject to western blotting. Representative blots (**a**) and summary data (**b**) are shown. n = 3-4, * p <0.05, **p <0.01 vs. WT, by ANOVA. (**c**) Representative pH 6.0-induced current traces at -60 mV of CHO cells transfected with WT and E/A mutant ASIC1a. (**d**) Summary data for peak currents of WT and E/A mutant ASIC1a expressed in CHO cells. n = 16–19, * p <0.05 vs. WT by unpaired t test. (**e**) E/A mutation decreased the expression of total ASIC1a as compared with WT, but not the surface expression (Surface/Total ratio).
