# Supplementary material for: Disruption of auto-inhibition underlies conformational signaling of ASIC1a to induce neuronal necroptosis
Source: Nat Commun. 2020 Jan 24;11:475. doi: 10.1038/s41467-019-13873-0 (PMC6981194; doi:10.1038/s41467-019-13873-0)
Supplement: Supplementary file 5 — Description of Additional Supplementary Files [file 41467_2019_13873_MOESM5_ESM.pdf]

Title: Supplementary Data 1
Description: The predicted peptides in MS.

Title: Supplementary Data 2
Description: Exact p value and related statistical methods
